# KYNU Expression Promotes Cisplatin Resistance in Esophageal Cancer

**DOI:** 10.7150/jca.93229

**Published:** 2024-03-11

**Authors:** Yu Lu, Xianyang Zhao, Mingliang Yuan, Ming Zhao, Kaisheng Liu, Miaomiao Zhang, Xiaoyan Qiu, Xuechun Yu, Xinliang Liu, Dongping Wei, Jun Xie, Zhongbin Cheng

**Affiliations:** 1 Department of Clinical Research Center, Xuyi People's Hospital, Affiliated Xuyi Hospital of Yangzhou University Medical College, Jiangsu, China.; 2 Department of pharmacy, Xuyi People's Hospital, Affiliated Xuyi Hospital of Yangzhou University Medical College, Jiangsu, China.; 3 Department of Gastroenterology, Xuyi People's Hospital, Affiliated Xuyi Hospital of Yangzhou University Medical College, Jiangsu, China.; 4 Department of Endocrinology, Xuyi People's Hospital, Affiliated Xuyi Hospital of Yangzhou University Medical College, Jiangsu, China.; 5 Department of Medical Research Center, The First Affiliated Hospital of Wenzhou Medical University, Zhejiang, China.; 6 Department of Inspection, Xuyi People's Hospital, Affiliated Xuyi Hospital of Yangzhou University Medical College, Jiangsu, China.; 7 Key Laboratory of Tropical Biological Resources of Ministry of Education, School of Pharmaceutical Sciences, Hainan University, Haikou, China.

**Keywords:** Cisplatin, Drug resistance, Esophageal cancer, Kynureninase, Tumor stemness

## Abstract

**Background:** Chemotherapy resistance is a barrier to effective cancer prognoses. Cisplatin (CDDP) resistance is a major challenge for esophageal cancer (EC) therapy. A deeper understanding of the fundamental mechanisms of cisplatin resistance and improved targeting strategies are required in clinical settings. This study was performed to identify and characterize a marker of cisplatin resistance in EC cells.

**Method:** KYSE140 and Eca-109 cells were subjected to escalating concentrations of cisplatin, resulting in the development of cisplatin-resistant KYSE140/CDDP and Eca-109/CDDP cell lines, respectively. RNA Sequencing (RNA-seq) was utilized to screen for the genes exhibiting differential expression between cisplatin-resistant and parental cells. Reverse transcription quantitative PCR was conducted to assess gene expression, and western blotting was employed to analyze protein levels. A sphere-formation assay was performed to validate tumor cell stemness. Cell counting kit-8 (CCK-8) experiments were conducted to confirm the sensitivity of cells to cisplatin. We examined the relationship between target genes and the clinicopathological features of patients with EC. Furthermore, the expression of target genes in EC tissues was evaluated via western blotting and fluorescence probe *in situ* hybridization (FISH).

**Results:** KYNU was upregulated in cisplatin-resistant EC cells (KYSE140/CDDP and Eca-109/CDDP cells) and in EC tissues compared to that in the respective parental cell lines (KYSE140 and Eca-109 cells) and non-carcinoma tissues. Downregulation of KYNU increased cell sensitivity to cisplatin and suppressed tumor stemness, whereas abnormal KYNU expression had the opposite effect. KYNU expression was correlated with the expression of tumor stemness-associated factors (SOX2, Nanog, and OCT4) and the tumor size.

**Conclusions:** KYNU may promote drug resistance in EC by regulating cancer stemness, and could serve as a biomarker and therapeutic target for EC.

## Introduction

Esophageal cancer (EC), one of the most aggressive gastrointestinal malignancies, is a contributor to global cancer-related mortality. The pathological classification of EC encompasses two primary subtypes: esophageal squamous cell carcinoma (ESCC) and esophageal adenocarcinoma 1. ESCC predominates in China, comprising approximately 90% of all EC cases 2. Despite advancements in screening, diagnosis, prevention, and treatment, the 5-year survival rates of ESCC patients remains are only 15%-25% 3. Individuals diagnosed with advanced or recurrent EC may have poor prognoses. Chemotherapy, with platinum drugs being the most effective, is widely regarded as the standard treatment for patients in advanced stages or with recurrence 4. Cisplatin, a small-molecule platinum compound initially characterized for its antibacterial properties and later recognized as an anticancer agent, has demonstrated promising outcomes in the treatment of advanced and recurrent gastrointestinal cancers 5. However, cisplatin resistance, which is a major contributing factor to treatment failure in patients with EC, significantly hampers the efficacy of this compound for the treatment of advanced or recurrent cancers. Therefore, it is crucial to elucidate the mechanisms underlying cisplatin resistance and identify novel biomarkers for predicting the drug response in EC patients.

Chemoresistance can be categorized into two type: inherent chemoresistance and chemoresistance acquired during drug treatment 6. Chemoresistance results from various factors that are influenced by the interplay among cancer cells, cancer stem-like cells (CSCs), and the tumor microenvironment. CSCs, also known as “tumor-initiating cells,” can reproduce all characteristics of the initial tumor mass, such as self-renewal and differentiation 7. These cells are considered crucial in explaining intratumor heterogeneity and become enriched after treatment, leading to tumor recurrence and metastasis. This indicates their significant role in the tumorigenicity, progression, and chemoresistance of various cancers 8, 9. Nanog, OCT4, and SOX2 serve as the transcription factors of CSCs, and were first identified in embryonic stem cells, which confer them with the capacity for pluripotent differentiation, meaning that they can regenerate the entire tumor mass 10. Several mechanisms, including enhanced drug efflux transporters, improved DNA repair machinery, elevated expression of anti-apoptotic proteins, and immune escape, contribute to the resistant phenotype of CSCs 11. These mechanisms can be targeted by inducing cell differentiation to inhibit self-renewal capabilities, promote apoptosis, block surface biomarkers, and target resistance mechanisms 12. Inhibiting CSC activity can thus effectively hinder tumor development.

The kynureninase (KYNU) gene, located on chromosome 2q22.2, encodes the kynurenine enzyme. This enzyme, which relies on pyridoxal-5′-phosphate, catalyzes the breakdown of L-kynurenine and L-3-hydroxykynurenine into anthranilic and 3-hydroxyanthranilic acids, respectively. KYNU plays a pivotal role in synthesis of NAD cofactors from tryptophan 13. It is associated with various inflammatory and cardiovascular diseases, encompassing syndromes such as cardiac, renal, and limb defects 14, 15. Elevated tumor KYNU mRNA expression in several types of cancers, including breast, colon, prostate, pancreatic cancer, stomach adenocarcinoma, and lung adenocarcinoma 16-18, is linked to the presence of an immunosuppressive tumor milieu and is a prognostic factor for poor overall survival. The cited research highlights the importance of KYNU as a tumor marker for immunosuppression and a prognostic indicator for survival across various types of cancer. Nonetheless, the role of KYNU in EC development remains ambiguous.

In this study, we aimed to identify a marker and potential therapeutic target for addressing cisplatin resistance in EC. Thus, we examined the genes differentially expressed between cisplatin-resistant cells and their parental cells to explore the functions of such target genes in mediating this phenotype in EC. We hypothesized that targeting these genes can potentially overcome tumor resistance and significantly improve tumor prognosis. To evaluate the validity of this hypothesis, we conducted a comprehensive set of experiments to explore the functions of these genes in mediating the cisplatin-resistant phenotype in EC.

## Materials and Methods

### Cell Culture

We chose two ESCC cell lines, namely Eca-109 and KYSE140 (Cell Bank of the Chinese Academy of Sciences, Shanghai, China), as the subjects for our study. Eca-109 and KYSE140 were cultivated in RPMI 1640 medium (KeyGen BioTECH, Nanjing,China), supplemented with 10% fetal bovine serum (YEASEN, Shanghai, China) and 100 U/mL penicillin/streptomycin (Gibco, NY, USA) and were maintained at 37 ℃ in a 5% CO_2_ incubator. The cisplatin-resistant cell lines Eca-109/CDDP and KYSE140/CDDP were established from Eca-109 and KYSE140 cells, respectively, through a 3-month incubation with a cisplatin concentration gradient. Subsequently, they were cultured in a medium containing 0.5 mg/ml cisplatin (15663-27-1, MedChemExpress, Shanghai, China). The culture medium was replenished every 2 days. HEK293FT cells were cultured in DMEM medium (KeyGen BioTECH, Nanjing, China) containing fetal bovine serum, penicillin, and streptomycin.

### RNA Sequencing (RNA-seq)

Following isolation and purification of the total RNA with the TRIzol reagent kit (15596018, Invitrogen, NY, USA), 1 μg of total RNA was used to extract Poly (A) RNA, which was then fragmented and subjected to two rounds of purification. The fragmented RNA was reverse-transcribed to generate cDNA, which was subsequently utilized to synthesize U-labeled second-stranded DNA. The U-labeled second-stranded DNA was then subjected to treatment with the heat-labile UDG enzyme (m0280, NEB, MA, USA) and amplified via PCR. The average insert size of the resultant cDNA library was 300 ± 50 base pairs. Subsequently, we conducted paired-end sequencing (PE150) with 2×150bp reads using Illumina NovaSeq™ 6000 platform (LC-Bio Technology Co., Ltd., Hangzhou, China). StringTie was employed for calculating the FPKM value to determine mRNA expression. Differentially expressed mRNAs were determined using a fold-change criteria (> 2 or < 0.5) and *p*-value < 0.05.

### Western Blotting (WB)

The proteins extracted from cultured cells and tissues were lysed with radioimmunoprecipitation assay buffer (P0013B, Beyotime, Shanghai, China), supplemented with protease inhibitor cocktail (P1005, Beyotime) and 1 mM phenylmethanesulfonyl fluoride (ST505, Beyotime). Following centrifugation at 13,500 × *g* and 4 ℃ for 15 min, the supernatant was collected. 30 μg protein was separated on 10% polyacrylamide gels and subsequently transferred to 0.22 μm PVDF membranes (Millipore, Darmstadt, Germany) after quantification using a BCA assay kit (P0012S, Beyotime). The membrane was blocked with 5% skimmed milk at room temperature for 1 h, followed by overnight incubation with the primary antibody at 4℃. Subsequently, it was treated with a horseradish peroxidase-conjugated secondary antibody and visualized using an enhanced ECL kit (P0018S, Beyotime). The images were recorded with the Clinx imaging system (Clinx Science Instruments Co., Ltd., China). The primary antibodies used were as follows: anti-KYNU (A6643, 1:2,000 dilution, ABclonal, Wuhan, China), anti-OCT4 (11263-1-AP, 1:1,000 dilution, Proteintech, Wuhan, China), anti-Nanog (14295-1-AP, 1:1,000dilution, Proteintech, Wuhan, China), anti-SOX2 (11064-1-AP, 1:1,000 dilution, Proteintech, Wuhan, China), anti-Bax (50599-2-Ig, 1:2,000 dilution, Proteintech, Wuhan, China), anti-Bcl-2 (12789-1-AP, 1:2,000 dilution, Proteintech, Wuhan, China), anti-GAPDH (10494-1-AP, 1:5,000 dilution, Proteintech, Wuhan, China), β-actin (A1978, 1:3,000 dilution, Sigma-Aldrich, St Louis, MO, USA). GAPDH and β-actin were utilized as the loading controls.

### RNA Extraction and Reverse Transcription Quantitative PCR Analysis

Cell RNA was isolated utilizing the Total RNA Kit I (R6834-02, Omega, Guangzhou, China). The RNA was reverse-transcribed into cDNA using the 5× All-In-One MasterMix (G492, abm®, BC, Canada), and subsequently subjected to reverse transcription quantitative PCR analysis with the BrightGreen 2× qPCR MasterMix-ROX (Master Mix-R, abm®, BC, Canada) and the ABI StepOne Plus instrument (Thermo Fisher Scientific^TM^, NY, USA). The thermocycling profile consisted of an initial denaturation at 95 ℃ for 10 min, followed by 40 cycles of denaturation at 95 ℃ for 15 s and annealing/extension at 60 ℃ for 1 min. Moreover, a melting curve analysis was performed. GAPDH served as the control gene. Expression levels of SESN3, TMEM30A, CCHCR1, HGD, SPINK6, HIGD1A, KYNU, SOD2, IL6ST, RLIM, CSRP1, CCND3, SOX2, Nanog, and OCT4 were quantified using the 2^-ΔΔCt^ method (Table 1).

### Transfection

shRNA targeting KYNU and plasmids for KYNU overexpression were designed to silence and enhance KYNU expression, respectively.The constructed plasmids were combined with pMD2G (encoding the VSV-G protein) and pPAX2 (encoding the lentivirus packaging vector) into HEK 293FT cells via transfection with Lipofectamine 8000 transfection reagent (C0533, Beyotime, Shanghai, China). After 48 h of culture, the cell culture supernatant was harvested. Two rounds of infection with the virus-containing supernatant were then performed, and the cells were treated with 1 μmol/mL bleomycin (60216ES60, YEASEN, Shanghai, China) for stable cell line selection. After 3 days of selection, the selected stable cell lines were collected for subsequent experiments.

### Cell Counting Kit-8 (CCK-8) Assay

Cell viability was determined with a CCK-8 assay kit (KGA9305-500, KeyGen BioTECH, Nanjing, China) at a density of 3.0×10^4^ cells/ml in 96-well plates. Following a 24 h incubation, EC cells were exposed to varying concentrations (0, 1, 2, 4, and 8 μM) of cisplatin for an additional 24 h. The absorbance at 450 nm was determined using a microplate reader (Multiskan FC, Thermo, NY, USA). Each concentration was assessed in triplicate. KYNU expression in Eca-109, KYSE140, and CDDP-resistant cells (Eca-109/CDDP and KYSE140/CDDP) was quantified via WB.

### Sphere-formation Assay

EC cells were harvested as single-cell suspensions through enzymatic dissociation, washed twice with 1× PBS, and then filtered using a 40 μm cell strainer. Subsequently, the cells were counted and seeded at a clonal density in 24-well ultra-low attachment plates (Corning, NY, USA) containing a specialized culture medium. The medium consists of DMEM/F12 (Gibco, NY, USA), 1×B27 supplement (17504044, Gibco, NY, USA), 20 ng/ml human recombinant epidermal growth factor (PHG0311, EGF, Gibco, NY, USA), 10 ng/ml heat-stable recombinant human bFGF (PHG0367, bFGF, Gibco, NY, USA), 0.4% BSA (30036578, Fraction V, Gibco, NY, USA), 0.5 mM HEPES (15630106, Gibco, NY, USA), and 1× penicillin-streptomycin (Gibco, NY, USA), which is suitable for tumor spheres formation. The formation of tumor spheres was allowed to proceed for 13-16 days, and microscopic evaluations were performed every 3 days. Images were captured using an inverted microscope (Olympus, Tokyo, Japan).

### Bioinformatic Analysis

The web-based tool Gene Expression Profiling Interactive Analysis (GEPIA) available at http://gepia.cancer-pku.cn/index.html was employed to conduct differential gene analysis within The Cancer Genome Atlas (TCGA), which comprises 182 tumor samples and 286 normal samples. Subsequently, we collected 11 pairs of cancer tissues with their corresponding adjacent tissues and examined KYNU expression using WB. The tissues were obtained from the Xuyi People's Hospital and stored in liquid nitrogen. We received approval from the Ethics Committee of Xuyi People's Hospital, China, and acquired informed consent from all patients.

### FISH

To detect KYNU expression, FISH was performed using a tissue array (Shanghai OUTDO Biotech, Shanghai, China) containing EC samples. First, paraffin sections were deparaffinized and dehydrated. Subsequently, they were subjected to repair and digestion. The tissue sections were treated with a pre-hybridization solution (Servicebio, Wuhan, China) and incubated at 37 ℃ for 1 h. Next, the solution was removed, and a hybridization solution (Servicebio, Wuhan, China) containing probes for KYNU was added and left to hybridize overnight at 40 ℃. The hybridization solution was rinsed, and the corresponding branch probe was added for hybridization. To prevent drying, 50 ml of 2× SSC (saline sodium citrate) was added to the bottom of the wet box. Afterward, the hybridization solution was poured off, and a new hybridization solution containing the signal probe (Cy3) was added, diluted at a ratio of 1:400, and incubated at 42 ℃ for 3 h. Next, DAPI counterstain was applied to the nucleus; the DAPI solution was dripped onto the slice, followed by an 8-min incubation at room temperature in the dark. The slice was then washed with running water and covered with an anti-fade mounting medium. Subsequently, microscopy was used for detection, and images were captured using fluorescence microscopy. CY3 emits red light at excitation and emission wavelengths of 510-560 and 590 nm, respectively.

### Statistical Analysis

All statistical analyses were conducted using the GraphPad Prism 7 software (GraphPad Inc., CA, USA). Group differences were evaluated using either Student's *t*-test or a one-way ANOVA, depending on the specific comparison. The chi-square test was utilized to investigate the correlation between KYNU expression and the clinicopathological parameters of EC patients. Survival curves of EC patients were constructed through the Kaplan-Meier method and analyzed with the log-rank test. *In vitro* data from at least three independent experiments are shown as mean ± SD; statistical significance was defined at *p* < 0.05.

## Results

### Establishment of Cisplatin-resistant Cell Lines

**Figure 1:**
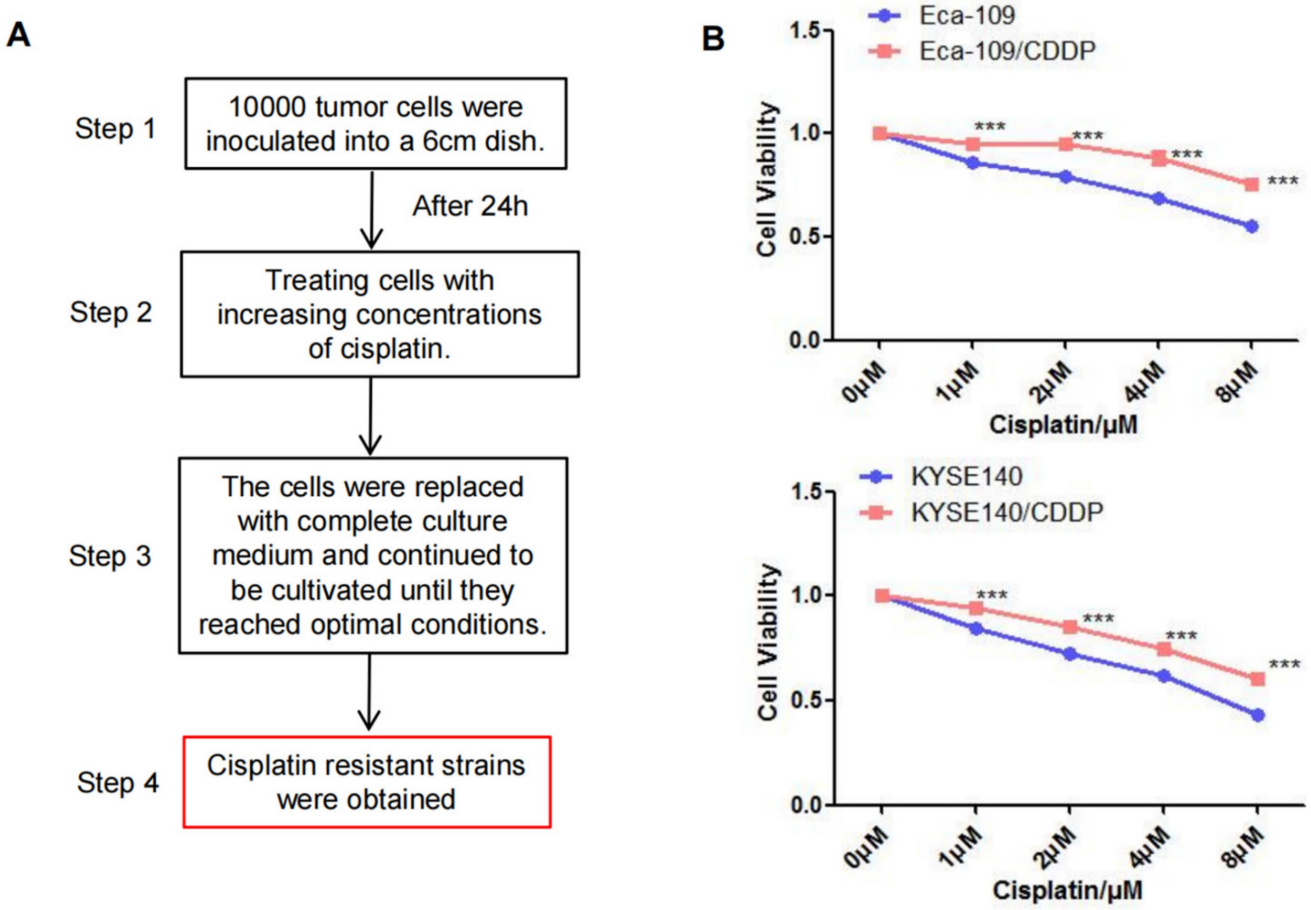
** Establishment of the cisplatin**-**resistant esophageal cancer cells.** The cisplatin-resistant cell lines (Eca-109/CDDP and KYSE140/CDDP) were generated by treating esophageal cancer cells (Eca-109 and KYSE140) with increasing concentrations of cisplatin (**A**). The resistance to cisplatin in these cell lines was confirmed using the CCK-8 assay (**B**), ***: *P* < 0.001.

After exposing parental Eca-109 and KYSE140 cells to a cisplatin concentration gradient for 3 months, we successfully established cisplatin-resistant cell lines (Eca-109/CDDP and KYSE140/CDDP; Figure 1A). Subsequently, Eca-109, Eca-109/CDDP, KYSE140, and KYSE140/CDDP cells were cultured and exposed to varying concentrations of cisplatin. The CCK-8 assay was utilized to measure cell viability inhibition. Cisplatin treatment resulted in a concentration-dependent viability inhibition in both the parental and cisplatin-resistant cell lines. However, the rate of inhibition of Eca-109 and KYSE140 cell viability was consistently higher than that of Eca-109/CDDP and KYSE140/CDDP cell viability (Figure 1B).

### Correlation between KYNU Expression and Cisplatin Resistance in EC

**Figure 2:**
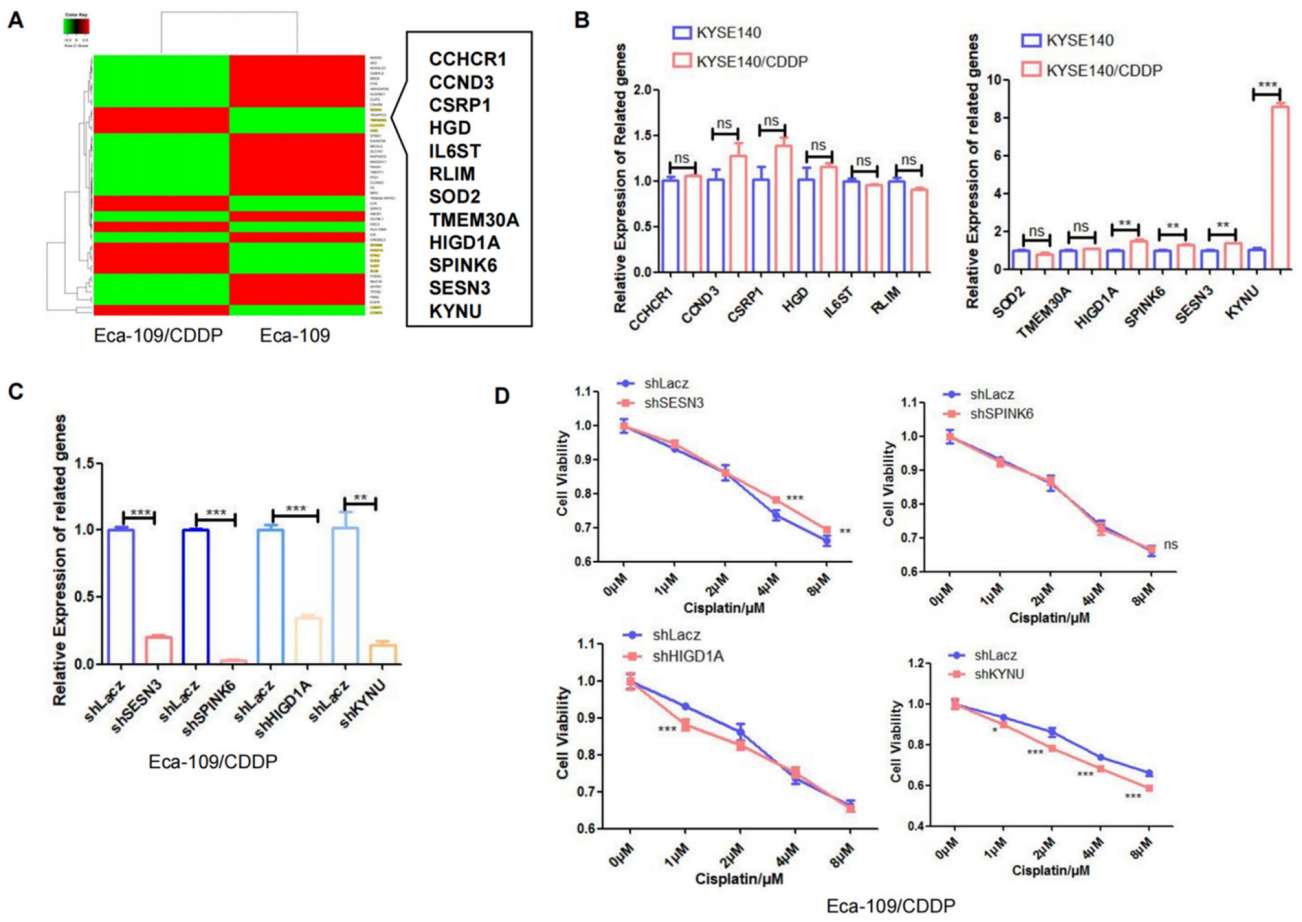
** KYNU expression is closely related to cisplatin resistance in esophageal cancer.** The esophageal cancer cell lines Eca-109 and Eca-109/CDDP were subjected to mRNA-seq analysis, which revealed the differential expression of 50 genes between these two cell lines (**A**). The expression levels of 12 genes, which were upregulated in Eca-109/CDDP cells, were investigated in KYSE140 and KYSE140/CDDP cells using real-time PCR (**B**). RNA interference technology was used to downregulate the SENS3, SPINK6, HIGD1A, and KYNU genes in Eca-109/CDDP cells (**C**). The aim of the experiment was to assess the correlation between the downregulation of genes and the sensitivity of Eca-109/CDDP cells to cisplatin using the CCK-8 assay (**D**), ns: *P*>0.05, *:*P*<0.05, **: *P*<0.01, ***: *P* < 0.001.

Next, RNA-seq analysis was utilized to identify differentially expressed genes between the cisplatin-resistant cell line Eca-109/CDDP and its parental counterpart Eca-109. The results revealed 12 genes, specifically SESN3, TMEM30A, CCHCR1, HGD, SPINK6, HIGD1A, KYNU, SOD2, IL6ST, RLIM, CSRP1, and CCND3, whose expression was increased in Eca-109/CDDP cells compared to that in Eca-109 cells (Figure 2A). We further corroborated these results by investigating the expression of these 12 genes in another cisplatin-resistant cell line (KYSE140/CDDP) and its parent cell line (KYSE140) using real-time PCR. Among these genes, SENS3, SPINK6, HIGD1A, and KYNU were significantly upregulated in KYSE140/CDDP cells relative to those in KYSE140 cells (Figure 2B). We validated the effect of downregulation of SENS3, SPINK6, HIGD1A, and KYNU on the sensitivity of cisplatin-resistant EC cell lines to cisplatin. The results showed that reduced KYNU expression significantly enhanced the effects of cisplatin on cisplatin-resistant EC cells (Figure 2C and 2D). These findings suggested a potential association between the upregulation of KYNU and cisplatin resistance in EC.

### Cisplatin Resistance in EC is Associated with Tumor Stemness

**Figure 3:**
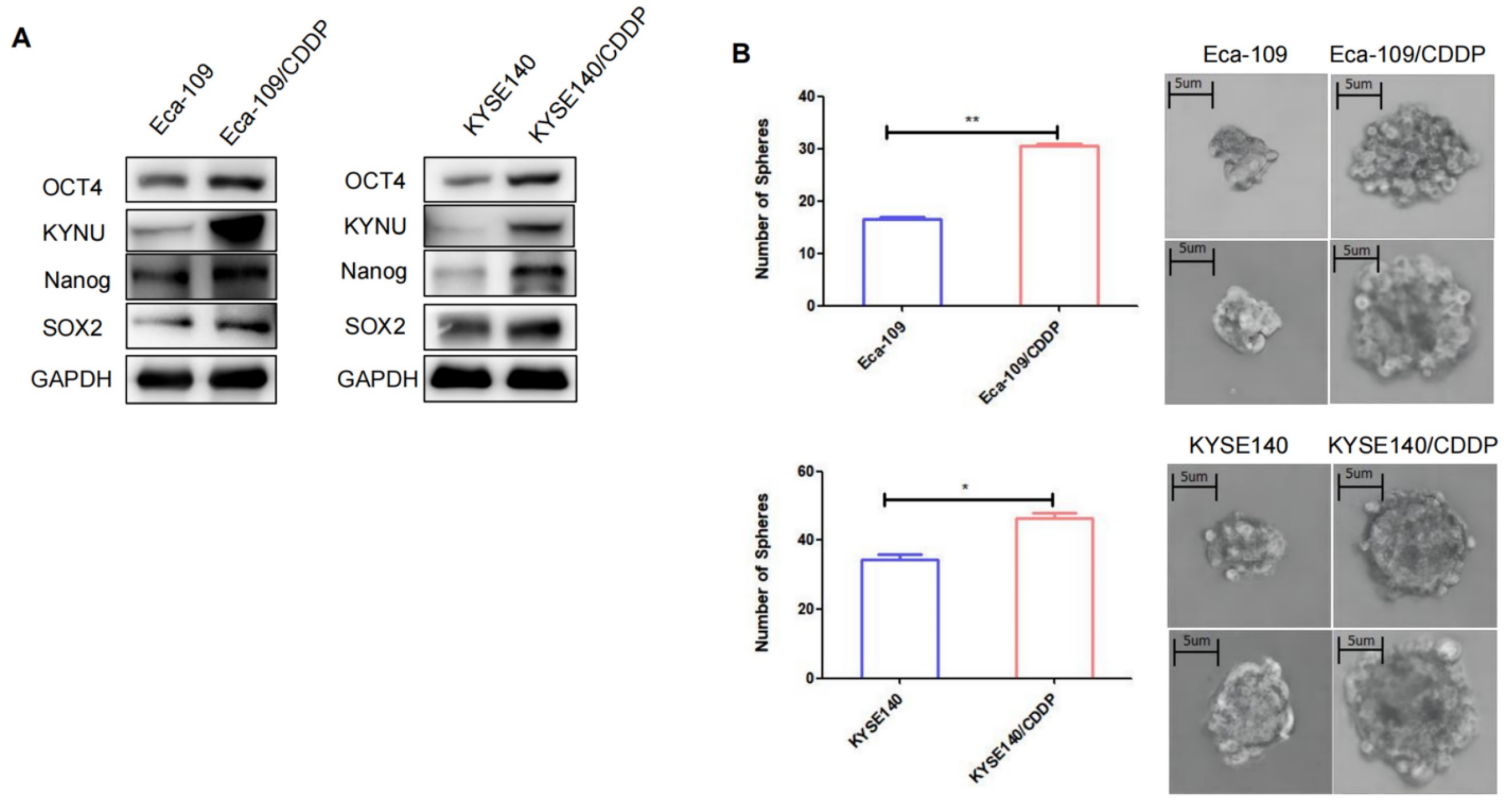
** Cisplatin resistance in esophageal cancer is correlated with tumor stemness.** To validate the expression of KYNU, SOX2, OCT4, and Nanog in cisplatin-resistant cell lines (Eca-109/CDDP and KYSE140/CDDP) and their parental cells (Eca-109 and KYSE140), we performed western blot analysis (**A**). Additionally, we investigated the sphere-forming ability of the two sets of cell lines using sphere-formation assays (**B**), *:*P*<0.05, **: *P*<0.01.

CSCs are a type of tumor cells exhibiting stem cell characteristics such as self-renewal and multidrug resistance 19. Additionally, CSCs can spontaneously form spherical three-dimensional structures (referred to as tumor spheres) in serum-free media containing growth factors and essential nutrients. Therefore, the sphere-forming capacity can be used to assess the stemness of tumor cells. The sphere-formation assay can generate and maintain highly tumorigenic CSCs. We compared the sphere-forming abilities of cisplatin-resistant strains and their parental cells using sphere-formation assays and validated the levels of tumor stemness-related factors in both cell types through WB. Results demonstrated that the levels of these factors, particularly SOX2, OCT4, and Nanog, were higher in the resistant strains than in their parental cells (Figure 3A). The cisplatin-resistant strains also exhibited higher sphere-forming capacity than their parental cells (Figure 3B).

### KYNU Regulates Cisplatin Resistance in EC by Controlling Tumor Stemness

**Figure 4:**
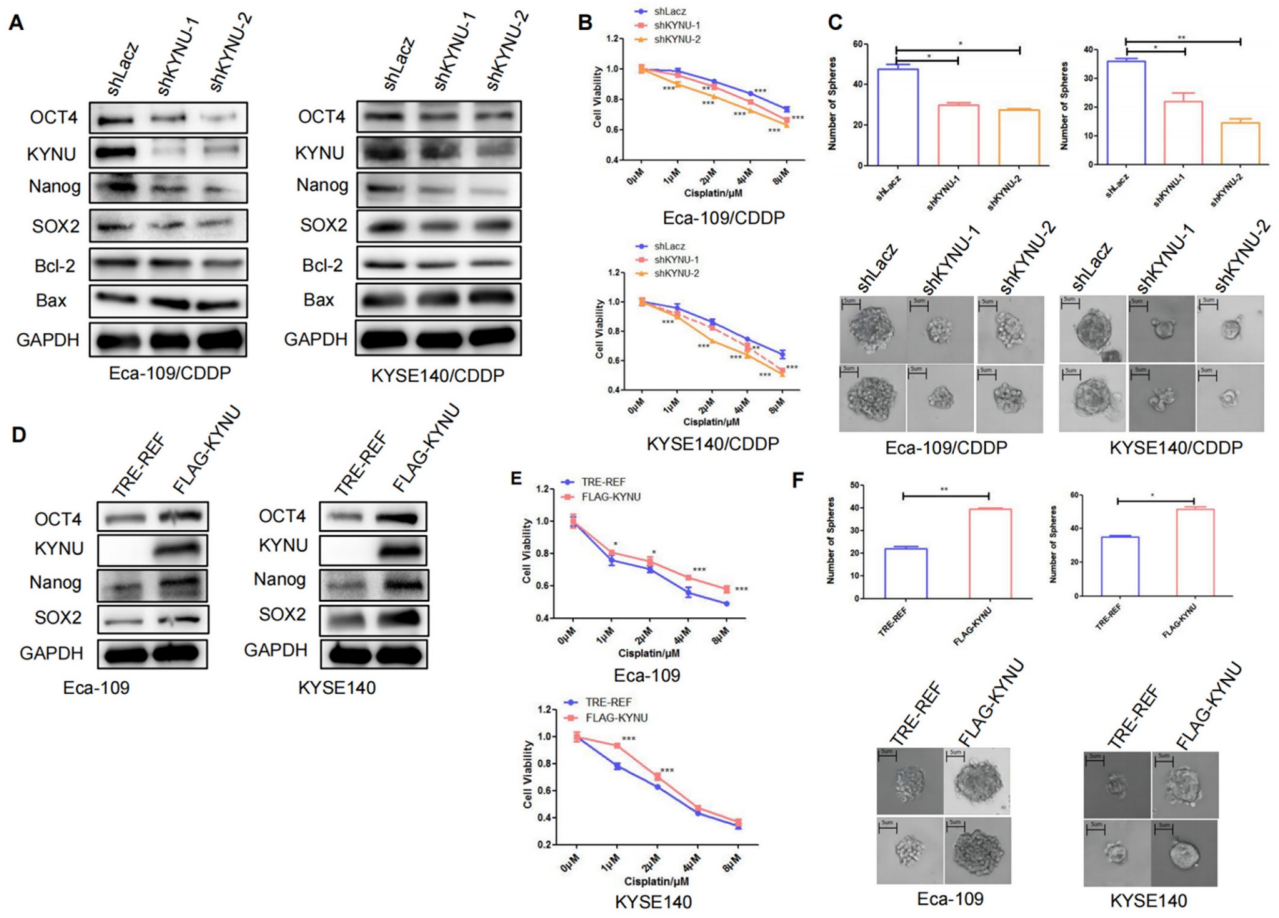
** KYNU regulates cisplatin resistance in esophageal cancer by controlling tumor stemness.** RNA interference and gene overexpression techniques were employed to inhibit or enhance the expression of KYNU in cisplatin-resistant cell lines (Eca-109/CDDP and KYSE140/CDDP) and their parental cells (Eca-109 and KYSE140). Protein levels of KYNU, SOX2, OCT4, Nanog, Bcl-2, and Bax were assessed using western blot analysis. Changes in KYNU expression affected the expression of a series of related proteins. Downregulation of KYNU led to the downregulation of various tumor stemness-related factors (SOX2, OCT4, Nanog) and the anti-apoptotic protein Bcl-2, while promoting the expression of the pro-apoptotic protein Bax (**A**). Then, we investigated the effects of KYNU expression on cisplatin sensitivity and tumor cell sphere-forming ability in EC using the CCK-8 and sphere-forming assays. The results showed that downregulation of KYNU led to an increase in sensitivity of Eca-109/CDDP cells to cisplatin and inhibited the formation of tumor cell spheres (**B** and **C**). However, upregulation of KYNU led to the opposite results (**D**-**F**), *:*P*<0.05, **: *P*<0.01, ***: *P* < 0.001.

We further explored the significance of elevated KYNU expression in cisplatin-resistant EC cell lines and its effect on tumor stemness. We first utilized RNA interference technology to suppress KYNU expression in cisplatin-resistant EC cell lines and established stable cell lines. Then, we validated its downregulation through WB. The findings indicated that the downregulation of KYNU resulted in reduced protein and mRNA levels of SOX2, OCT4, and Nanog (Figure 4A and Supplementary Figure S1), while also resulting in reduced Bcl-2 expression and elevated Bax expression. We also conducted CCK-8 and sphere-formation assays to validate the influence of KYNU suppression on cisplatin resistance and the sphere-forming ability of drug-resistant EC cell lines. The results showed that a reduction in KYNU expression overcame the resistance of drug-resistant cell lines to cisplatin and reduced the sphere-forming ability of tumor cells (Figure 4B and C). These findings indicate that downregulating KYNU expression can inhibit tumor stemness and regulate cisplatin resistance in cisplatin-resistant EC cell lines.

Next, to clarify the relationship of KYNU with tumor stemness and cisplatin resistance, we employed molecular cloning techniques and gene overexpression technology to manipulate the expression of KYNU in EC cells, which resulted in the establishment of stable cell lines overexpressing KYNU (Figure 4D). Through multiple experiments, we validated the effects of increased KYNU expression on the stemness of parental tumor cells and their susceptibility to cisplatin. In contrast to the results observed with KYNU downregulation, the upregulation of KYNU expression in EC cells promoted the expression of SOX2, OCT4, Nanog, and Bcl-2 while inhibiting that of Bax (Figure 4D). Upregulation of KYNU expression resisted the cytotoxic effects of cisplatin on tumor cells and enhanced tumor cell sphere formation (Figure 4E and F).

### Upregulation of KYNU in Human EC Tissue

**Figure 5:**
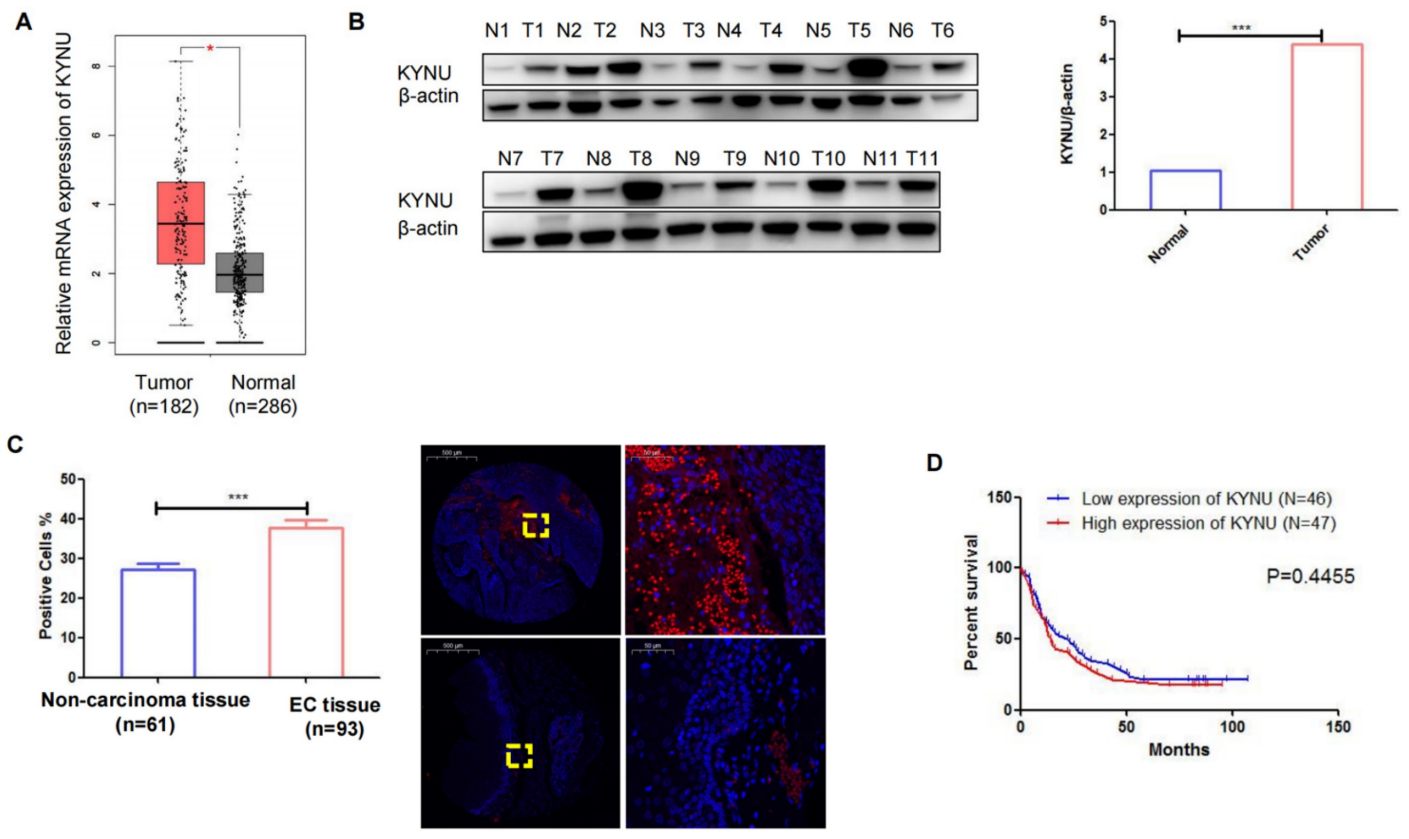
** KYNU is upregulated in esophageal cancer (EC) tissues.** mRNA levels of KYNU in EC tissues and normal tissues samples in TCGA database (**A**). Protein expression of KYNU in EC tissue (T) and normal tissues (N) were assessed via western blotting (**B**). Gene expression of KYNU in EC tissues and non-carcinoma tissues was assessed via fluorescence probe *in situ* hybridization (**C**). Kaplan-Meier survival curves showing that EC patients with high KYNU gene expression (n = 47) had an overall survival similar to that of patients with low KYNU expression (n = 46) (**E**) *:*P*<0.05, **: *P*<0.01, ***: *P* < 0.001.

TCGA data demonstrated a substantial elevation in the mRNA levels of KYNU in EC tissues compared to adjacent normal tissues (Figure 5A). Subsequently, we conducted western blot analysis to examine KYNU expression in 11 pairs of cancer tissues and adjacent noncancerous tissues, which revealed a marked increase in KYNU protein expression in EC tissues relative to that in adjacent normal tissues (Figure 5B and C). FISH was employed to quantify KYNU expression in 93 EC tissue samples (Table 2). The results showed a higher number of positive cells in cancerous tissues than in the adjacent normal tissues (Figure 5D). Additionally, we examined the association between KYNU expression and clinicopathological features in EC. According to the median KYNU expression in tumor specimens, the 93 tumor samples were categorized into high- and low-expression groups. The analysis indicated a significant association between KYNU expression and tumor size, but no correlation with age, sex, or lymph node metastasis (Table 3). Kaplan-Meier survival analysis further demonstrated comparable overall survival among patients with EC exhibiting high and low KYNU expression (Figure 5E).

## Discussion

EC is a leading contributor to cancer-related mortality, and its incidence is increasing worldwide. Surgical resection, especially esophagectomy, is the standard treatment for early-stage EC patients. However, approximately 50% of patients gradually experience local or systemic recurrence as the disease progresses after resection, resulting in unfavorable outcomes 20. Multimodal regimens for EC often involve cisplatin-based chemotherapy. However, low sensitivity or chemoresistance lead to a poor prognosis and increase the financial burden on patients 21, 22. Understanding cisplatin resistance mechanisms and improving sensitivity to this agent are therefore important for the treatment of EC. This study shows a significant male preponderance among patients and the upregulation of KYNU in EC tissue and its correlation with chemotherapy resistance in this disease. The inhibition of KYNU expression in EC cells was found to suppress tumor stemness and stem cell characteristics, consequently regulating cisplatin resistance in cisplatin-resistant strains. Furthermore, the expression of KYNU is correlated with tumor size. These findings suggest that KYNU might play a crucial role in EC therapy.

KYNU is essential for the degradation of kynurenine. The KYNU pathway constitutes the main pathway for tryptophan metabolism in most mammalian cells, leading to the degradation of kynurenine to 3-hydroxyanthranilic acid. This process reduces Foxp3 expression and enhances the inflammatory response 14. KYNU is associated with numerous diseases, including hypertension, inflammation, cardiovascular diseases, and various types of cancers through different pathways. For example, KYNU expression is elevated in psoriasis and is positively correlated with the severity of the condition and the level of inflammation 14. KYNU depletion suppresses the motility and growth of cutaneous squamous cell carcinoma (CSCC) cells through the inhibition of the PI3K/AKT pathway 23. The overexpression of KYNU markedly inhibits the proliferation of breast cancer cells and the growth of tumors in mice 24. These findings suggest that the patterns and effects of KYNU expression may vary with the specific disease. However, despite its association with noncancerous diseases and certain types of cancer, the role of KYNU in EC previously remained unknown.

We investigated the potential role of KYNU in cisplatin-resistant EC cell lines. There was a significant elevation in KYNU expression in cisplatin-resistant cell lines compared to the parental cells. Elevated KYNU expression was observed in human EC tissues relative to adjacent normal tissues. To investigate the involvement of KYNU in chemotherapy resistance, stable cell lines with either reduced or increased KYNU expression were established using a lentiviral packaging system. Subsequent CCK-8 assays were conducted to assess how the altered expression of KYNU affected the sensitivity of EC cells to cisplatin.

The findings indicated that the suppression of KYNU enhanced the sensitivity of cisplatin-resistant strains and impeded the formation of tumor cell spheres. In contrast, KYNU overexpression conferred resistance in EC cells and promoted tumor cell sphere formation. Mechanistic studies further demonstrated that KYNU knockdown affected the expression of several associated proteins, including SOX2, Nanog, OCT4, Bax, and Bcl-2. Specifically, the loss of KYNU expression reduced the expression of SOX2, Nanog, OCT4, and Bcl-2 and increased that of Bax; conversely, the upregulation of KYNU yielded the opposite effects. SOX2, Nanog, and OCT4 are pivotal factors associated with CSCs. CSCs possess self-renewal and differentiation capabilities and are highly tumorigenic. Their abundance strongly correlates with the advancement and invasion of cancer, as well as heightened resistance to standard cancer therapies including chemotherapy and radiotherapy 25. Thus, targeting CSCs is considered an innovative approach for clinical cancer therapy 26. KYNU influences the expression of SOX2, Nanog, and OCT4 at both the mRNA and protein levels, displaying a positive correlation. It may modulate the protein expression by regulating the mRNA levels of these three stem cell-related factors.

This article provides original insights into basic research on KYNU and establishes a new theoretical foundation for identifying specific targets for the diagnosis of EC and the implementation of gene therapy. KYNU expression was found to be positively correlated with both self-renewal ability and chemotherapy resistance in EC cell lines. These results show the link between KYNU and EC, and indicate the potential of KYNU as a therapeutic target. This study however represents a preliminary exploration, and more evidence is required for reaching a strong conclusion. Future studies should explore the role of KYNU in animal models and the signaling pathways downstream of KYNU in cancer, and reaffirm the influence of KYNU on the prognosis and clinical features of esophageal cancer patients through a more extensive sample size.

## Supplementary Material

Supplementary figure.

## Figures and Tables

**Table 1:** The sequences of the primer for genes.

Genes	Forward (5' to 3')	Reverse (5' to 3')
SESN3	CTGGGAAAATCATGGGTTCTCC	GCATGGTTGTGTCAACATCCT
TMEM30A	CAAAACCATCGTCGTTACGTGA	GTTGGCAATAGCTCCACAAGG
CCHCR1	CTGAAGTGGAAACTCGGCTG	GAATCTGGCGTAAGGAGACCA
HGD	AGCTCTCAGGATCGGCTTTCA	CGTCAATGGATTCAAAGGGCTT
SPINK6	TGACTGTGGTGAGTTCCAGGA	CCACTTTTCACTATGGCCTTACA
HIGD1A	AAGAGGCACCATTCGTACCC	ACCAACAGTCATTGCTCCTACA
KYNU	GTCACAACTACAACTTCACGGA	CCCCACTGAACAGGATCACTG
SOD2	TTTCAATAAGGAACGGGGACAC	GTGCTCCCACACATCAATCC
IL6ST	CGGACAGCTTGAACAGAATGT	ACCATCCCACTCACACCTCA
RLIM	GCAGTGAGTCGGACTAATCCA	CACTAGAACGTCTTGCAGATGG
CSRP1	ATTTACTGCAAGTCCTGCTACG	GCTTCCTCGTGCTTGATACCC
CCND3	TACCCGCCATCCATGATCG	AGGCAGTCCACTTCAGTGC
SOX2	TTTGTGGGCCTGAAGAAAACT	AGGGCTGTCCTGAATAAGCAG
Nanog	GCCGAGTGGAAACTTTTGTCG	GGCAGCGTGTACTTATCCTTCT
OCT4	CCTGAAGCAGAAGAGGATC	CGTTTGGCTGAATACCTT
GAPDH	GAGAAGTATGACAACAGCCTCAA	GCCATCACGCCACAGTTT

**Table 2:** Clinicopathological data of the 93 esophageal cancer patients included in the present study.

Clinicopathological parameter	Patient, n (%)
Gender	
Male	69 (74.19)
Female	24 (25.80)
Age, years	
<66	43 (39.78)
≥66	50 (60.22)
Tumor size	
<5 cm	45 (48.38)
≥5 cm	48 (51.61)
Pathological grading	
I	6 (6.45)
I-II	20 (21.51)
II	47 (50.54)
II-III	11 (11.83)
III	9 (9.68)
Tumor stage	
T1	8 (8.60)
T2	15 (16.13)
T3	70 (75.27)
Lymph node involvement	
N0	43 (46.24)
N1	30 (32.26)
N2	15 (16.13)
N3	5 (5.38)

**Table 3:** Correlation between KYNU and clinicopathological parameters of 93 esophageal cancer patients.

Parameter	KYNU expression level		*p*-value
	Low (n=46)	High (n=47)	
Gender			
Male	32	37	0.3506
Female	14	10	
Age, years			
<66	20	23	0.6749
≥66	26	24	
Tumor size			
<5 cm	28	17	0.0227*
≥5 cm	18	30	
Tumor stage			
T1	4	4	0.9405
T2	8	7	
T3	34	36	
Lymph node involvement			
N0	23	20	0.8016
N1	15	15	
N2	6	9	
N3	2	3	
