# Supplementary figures and images for: KYNU Expression Promotes Cisplatin Resistance in Esophageal Cancer
Source: J Cancer. 2024 Mar 11;15(9):2475–85. doi: 10.7150/jca.93229 (PMC10988315; doi:10.7150/jca.93229)

Supplementary figure S1

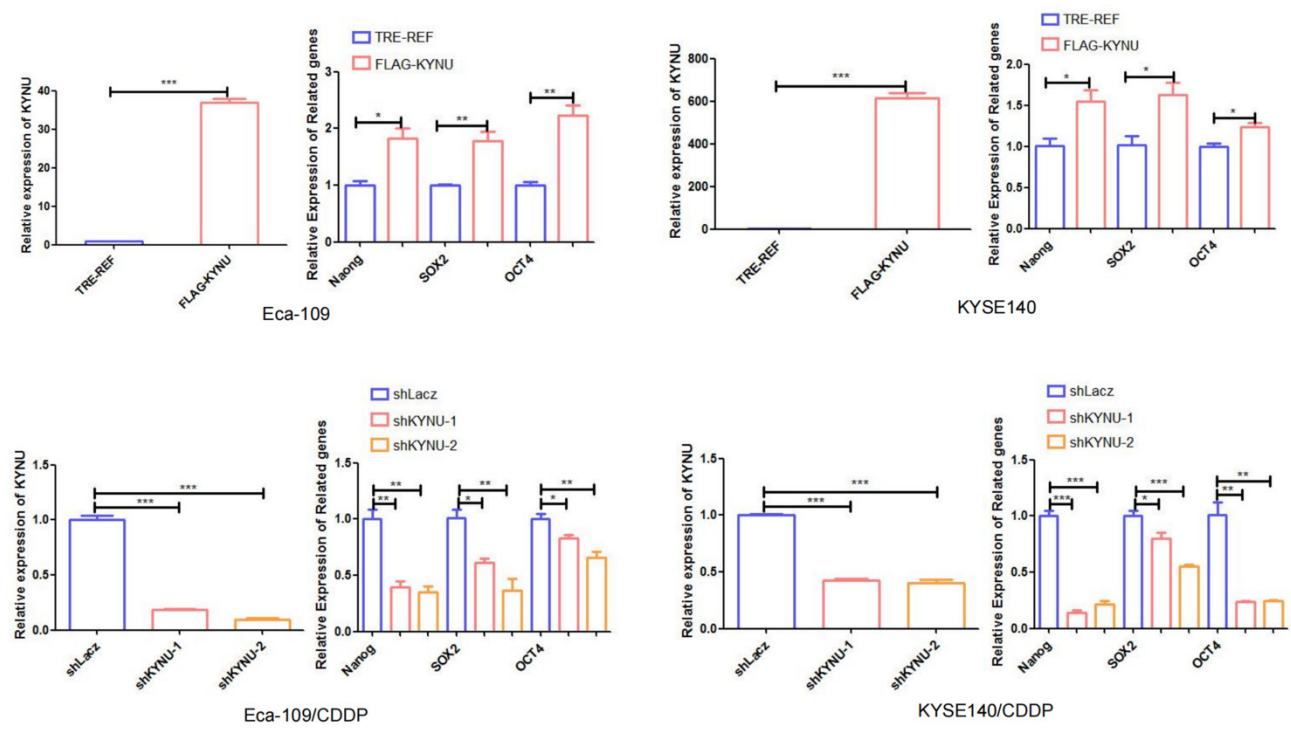

Supplement: Supplementary file 1 — Supplementary figure. [file jcav15p2475s1.pdf]
